# Anti-Diabetic Therapy and Heart Failure: Recent Advances in Clinical Evidence and Molecular Mechanism

**DOI:** 10.3390/life13041024

**Published:** 2023-04-16

**Authors:** Chih-Neng Hsu, Chin-Feng Hsuan, Daniel Liao, Jack Keng-Jui Chang, Allen Jiun-Wei Chang, Siow-Wey Hee, Hsiao-Lin Lee, Sean I. F. Teng

**Affiliations:** 1 Division of Cardiology, Department of Internal Medicine, National Taiwan University Hospital Yunlin Branch, Yunlin 640, Taiwan; 2 Division of Cardiology, Department of Internal Medicine, E-Da Hospital, I-Shou University, Kaohsiung 824, Taiwan; 3 Division of Cardiology, Department of Internal Medicine, E-Da Dachang Hospital, I-Shou University, Kaohsiung 824, Taiwan; 4 School of Medicine, College of Medicine, I-Shou University, Kaohsiung 840, Taiwan; 5 Graduate Institute of Medical Genomics and Proteomics, College of Medicine, National Taiwan University, Taipei 100, Taiwan; 6 Biological Programs for Younger Scholar, Academia Sinica, Taipei 115, Taiwan; 7 Department of Internal Medicine, National Taiwan University Hospital, Taipei 100, Taiwan; 8 Department of Cardiology, Ming-Sheng General Hospital, Taoyuan 330, Taiwan

**Keywords:** anti-diabetic therapy, heart failure, randomized clinical trials, molecular mechanism, sodium-glucose co-transporter-2 inhibitors

## Abstract

Diabetic patients have a two- to four-fold increase in the risk of heart failure (HF), and the co-existence of diabetes and HF is associated with poor prognosis. In randomized clinical trials (RCTs), compelling evidence has demonstrated the beneficial effects of sodium-glucose co-transporter-2 inhibitors on HF. The mechanism includes increased glucosuria, restored tubular glomerular feedback with attenuated renin–angiotensin II–aldosterone activation, improved energy utilization, decreased sympathetic tone, improved mitochondria calcium homeostasis, enhanced autophagy, and reduced cardiac inflammation, oxidative stress, and fibrosis. The RCTs demonstrated a neutral effect of the glucagon-like peptide receptor agonist on HF despite its weight-reducing effect, probably due to it possibly increasing the heart rate via increasing cyclic adenosine monophosphate (cAMP). Observational studies supported the markedly beneficial effects of bariatric and metabolic surgery on HF despite no current supporting evidence from RCTs. Bromocriptine can be used to treat peripartum cardiomyopathy by reducing the harmful cleaved prolactin fragments during late pregnancy. Preclinical studies suggest the possible beneficial effect of imeglimin on HF through improving mitochondrial function, but further clinical evidence is needed. Although abundant preclinical and observational studies support the beneficial effects of metformin on HF, there is limited evidence from RCTs. Thiazolidinediones increase the risk of hospitalized HF through increasing renal tubular sodium reabsorption mediated via both the genomic and non-genomic action of PPARγ. RCTs suggest that dipeptidyl peptidase-4 inhibitors, including saxagliptin and possibly alogliptin, may increase the risk of hospitalized HF, probably owing to increased circulating vasoactive peptides, which impair endothelial function, activate sympathetic tones, and cause cardiac remodeling. Observational studies and RCTs have demonstrated the neutral effects of insulin, sulfonylureas, an alpha-glucosidase inhibitor, and lifestyle interventions on HF in diabetic patients.

## 1. Introduction

Diabetic patients are associated with a higher risk of heart failure (HF) than those with euglycemia. Various randomized studies showed a significant bidirectional connection between diabetic patients and heart disease in past decades. Hyperglycemia is established as an independent risk factor for ischemic heart disease (IHD) through several mechanisms that lead to vascular damage from long-term hyperglycemia.

However, the reduction in hospitalized heart failure (HHF) was an unexpected finding in clinical trials of sodium-glucose co-transporter-2 (SGLT2) inhibitors. Although SGLT2 was known for over 25 years, the pharmacological and physiological effects of the transporter at a molecular level were not extensively explored until very recently. The most surprising fact was that the benefits are comparable to or even better than those achieved by recently approved drugs for HF. The benefits even persisted independently of blood sugar levels. The mechanisms were not well understood, and further trials of these drugs were conducted vigorously in recent years.

The prevalence of type 2 diabetes (T2D) has now reached a pandemic scale. In 1972, postmortem pathological findings from diabetic patients showed evidence ofHF without coronary arterial or valvular disease [1]. The Framingham study showed that the development of HF is two times more likely in men with diabetes and five times more likely in women with diabetes after adjustment for other risk factors, including age, coronary heart disease, and hypertension [2]. It is also known that approximately 50% of cases of diabetes mellitus (DM) suffer from HF with preserved ejection fraction (HFpEF) [3]. Therefore, DM has been perceived as a factor responsible for HF.

Observational studies have revealed a two- to four-fold increase in the risk of HF in patients with hyperglycemia [2,4]. Moreover, studies have reported that the incidence of HF in diabetic patients is significantly correlated with HbA1c levels [5]. HF is the most common first presentation of cardiovascular (CV) disease in patients with T2D [6]. Interactions between numerous pathways contribute to myocardial remodeling and cardiomyocyte dysfunction, resulting in HF. Different classes of anti-diabetic agents have shown different impacts on incident HF.

The heart has a high rate of energy consumption to maintain the cardiac contractility that delivers blood and oxygen to all other organs. Alterations in cardiac energetic metabolism contribute to the impediment of heart function without reference to coronary vascular lesions. Normally, cardiac energy is mainly obtained from fatty acid oxidation (FAO). However, under stress conditions, FAO may be reduced, and glucose utilization increased [7]. The adaptations in both glycolysis and mitochondrial oxidative metabolism in the diabetic heart induce the development of HF [8].

The prevalence of HF in patients with DM is greater than that in the general population. In DM patients, a 1% increase in HbA1c is associated with an 8% increased risk for HF [9].

Long-standing metabolic and functional alterations of hyperglycemia induce the glycation of numerous macromolecules that result in decreased elasticity of the vessel walls and myocardial dysfunction, which ultimately leads to irreversible structural changes [8].

Although the evidence has been solidified by large-scale double-blind randomized controlled trials, the true physiological and pharmacological mechanisms are still under debate. Through a deeper understanding of the mechanisms of anti-diabetic drugs on HF, we may discover more insights regarding the development of diabetic HF.

## 2. Anti-Diabetic Drugs and Heart Failure: Recent Progress from Clinical Trials

### 2.1. Sodium-Glucose Co-Transporter-2 Inhibitor (SGLT2 Inhibitors)

SGLT-2 inhibitors are a novel class of anti-diabetic agents that inhibit glucose reabsorption in renal proximal convoluted tubules. SGLT2 inhibitors offer an insulin-independent mechanism of action, and multiple landmark clinical trials support their effectiveness in reducing blood glucose levels. In addition, SGLT2 inhibitors have shown favorable effects on body weight (BW), blood pressure (BP), lipid profile, arterial stiffness, and endothelial function. More impressively, they have demonstrated significant cardioprotective and renoprotective effects.

#### 2.1.1. Prevention of HF in Diabetic Patients

The Swedish HF registry has shown a markedly reduced median survival of 3.5 years in patients with HF and T2D, compared with 4.6 years in those with HF alone [10]. Thus, the prevention of HF in patients with T2D is a top priority. Several large-scale CV outcome trials of SGLT2 inhibitors in individuals with T2D (Table 1) consistently showed an early reduction in incident HF in approximately 85–90% of patients without HF at baseline, and the effect was independent of glucose lowering per se [11,12,13]. The EMPA-REG OUTCOME trial showed that empagliflozin reduced the composite of CV mortality or HHF by 34%, and HHF by 35% in patients with T2D and CV disease. The benefit was consistent in patients with and without baseline HF [14]. In the CANVAS trial, treatment with canagliflozin reduced CV death or HHF, with a hazard ratio (HR) of 0.78. Treatment with canagliflozin in patients with a prior history of HF showed a greater benefit compared with those without baseline HF (HR: 0.61 vs. 0.87; *p* for interaction = 0.021) [15]. In the DECLARE–TIMI 58 trial, dapagliflozin reduced the rate of CV death or HHF by 17%, and HHF by 27% [13]. Treatment with dapagliflozin showed a greater reduction in CV death or HHF in patients with HF with reduced ejection fraction (HFrEF) than in those without HFrEF (HR: 0.62 vs. 0.88; *p* for interaction = 0.046) [16]. The VERTIS CV trial showed that ertugliflozin did not significantly reduce first HHF or CV death (HR, 0.88 (95% confidence interval (CI), 0.75–1.03)), but significantly reduced the risk for first HHF by 30%, which was not influenced by previous HF [17,18]. SGLT2 inhibitors are therefore recommended for preventing HF in patients with T2D in both American and European guidelines for the management of HF [19,20].

In patients with diabetic kidney disease (DKD), there are several trials confirming the benefit of SGLT2 inhibitors (Table 1), in addition to renin–angiotensin system (RAS) blockade, in decreasing HF events [21,22,23]. In the CREDENCE trial, all patients had DKD with an estimated glomerular filtration rate (eGFR) of 30 to <90 mL/min/1.73 m^2^ and a urinary albumin-to-creatinine ratio (UACR) of >300 to 5000 mg/g, and were treated with RAS inhibitors. Canagliflozin was associated with a 31% lower risk of CV death or HHF and a 39% lower risk of HHF [21]. The efficacy of canagliflozin was consistent regardless of prior history of HF at baseline [24]. The SCORED trial showed that in patients with T2D, chronic kidney disease (CKD) (eGFR, 25 to 60 mL/min/1.73 m^2^), and risks for CV disease, sotagliflozin resulted in a 26% reduction of the risk of the primary endpoint of the composite of the total number of deaths from CV causes, HHF, and urgent visits for HF. The effect of sotagliflozin was similar in patients with or without HF [22]. The DAPA-CKD trial enrolled patients with an eGFR of 25 to 75 mL/min/1.73 m^2^ and a UACR of 200 to 5000 mg/g. The composite of death from CV causes or HHF was decreased with dapagliflozin by 29% versus placebo. The effect was similar in participants with and without T2D [23], as well as in patients with and without HF [25]. The benefit was largely driven by a reduction of 49% in HHF, which was similar in patients with and without HF [25]. The EMPA-KIDNEY studied patients with CKD who had an eGFR of at least 20, but less than 45 mL/min/1.73 m^2^, or an eGFR of at least 45, but less than 90 mL/min/1.73 m^2^ plus a UACR of at least 200 mg/g. There were, however, no significant between-group differences with respect to the composite outcome of HHF or CV death [26].

#### 2.1.2. Treatment of Heart Failure in Diabetic Patients

SGLT2 inhibitors are the only class of anti-diabetic drugs proven to be beneficial in the management of patients with established HF, either chronic HF with reduced, mildly reduced, or preserved ejection fraction, or acute decompensated HF. In patients with symptomatic HF with a left ventricular ejection fraction (LVEF) of 40% or less, the DAPA-HF trial demonstrated that over a median of 18.2 months, dapagliflozin reduced the primary outcome (a composite of worsening HF, hospitalization, an urgent visit resulting in intravenous therapy for HF, or CV death), the first worsening HF event, death from CV causes, and death from any cause by 26%, 30%, 18%, and 17%, respectively [27]. Notably, dapagliflozin led to a 32% decrease in new-onset diabetes [28]. The EMPEROR-Reduced trial showed the benefit of empagliflozin by reducing the primary outcome (a composite of CV death or HHF) by 25% in patients with HFrEF. Empagliflozin treatment reduced the risk of HHF by 30% [29].

In patients with New York Heart Association (NYHA) functional classes II-IV HFpEF, empagliflozin reduced the combined risk of CV death or HHF by 21% over 26.2 months, mainly related to a lower risk of HHF, in the EMPEROR-Preserved trial. Empagliflozin lowered the total number of HHF by 27% [30]. In the DELIVER trial, dapagliflozin reduced the combined risk of worsening HF, defined as either an unplanned HHF or an urgent visit for HF, or CV death by 18% over a median of 2.3 years [31]. The beneficial effects of SGLT2 inhibitors on the primary outcomes were consistent in patients independent of the presence of diabetes [28,29,30,31].

The SOLOIST-WHF trial examined the effect of sotagliflozin in patients with T2D who were recently hospitalized for worsening HF. The treatment was initiated immediately after an episode of HF (48.8% before discharge and 51.2% in a median of 2 days after discharge). Sotagliflozin lowered the total number of deaths from CV causes and hospitalizations and urgent visits for HF by 33%, and this benefit was consistent in participants stratified according to LVEF (<50% or ≥50%) [32].

The EMPULSE trial enrolled 530 patients with a primary diagnosis of acute de novo or decompensated chronic HF regardless of LVEF. The patients were randomized in hospital when clinically stable, with a median time from hospital admission to randomization of only 3 days. More patients treated with empagliflozin experienced a clinical benefit, defined as a hierarchical composite of death from any cause, number of HF events, and time to first HF event, or a 5-point or greater difference in the change from baseline in the Kansas City Cardiomyopathy Questionnaire Total Symptom Score at 90 days, compared with placebo. Clinical benefit was observed for both acute de novo and decompensated chronic HF and was observed regardless of LVEF or the presence or absence of diabetes, indicating that the initiation of empagliflozin in patients hospitalized for acute HF is feasible [33].

The CHIEF-HF trial randomized 476 HF patients, regardless of EF or diabetes status, to receive 100 mg of canagliflozin or placebo, and was conducted in a completely remote fashion without in-person interactions between doctor and patient. The primary outcome—a change in the Kansas City Cardiomyopathy Questionnaire Total Symptom Score at 12 weeks—was 4.3 points higher with canagliflozin than with placebo, demonstrating a significant improvement in the symptom burden with canagliflozin. The effects were similar in participants with HFpEF or HFrEF and in participants with and without diabetes. However, the study was not designed or powered to examine clinical events [34]. The evidence mentioned above suggests that SGLT2 inhibitors are an all-encompassing therapy for HF, and they can be initiated in all patients with HF who do not have contraindications and at any point and time of contact [35]. Accordingly, the Consensus Report of the American Diabetes Association recommends prioritizing the use of SGLT2 inhibitors in individuals with stage B HF and in all patients with stages C or D HF, without taking LVEF into consideration [9].

### 2.2. Glucagon-like Peptide Receptor Agonist (GLP-1 RA)

GLP-1 receptor agonists (also known as GLP-1 agonists, incretin mimetics, or GLP-1 analogs) stimulate insulin secretion through the effect of incretin. They are structurally classified into two categories: human GLP-1 backbone agents (known as glutides) and exendin-4 backbone agents (known as enatides). GLP-1 RAs delay gastric emptying and inhibit the production of glucagon if the level of blood sugar rises. Furthermore, GLP-1 receptor agonists can reduce pancreatic beta-cell apoptosis while promoting their proliferation. In addition, semaglutide and high-dose liraglutide are approved by the Food and Drug Administration (FDA) as pharmacologic treatments for overweight patients with comorbidities.

In patients with T2D and established CV disease enrolled in the Harmony Outcomes trial, treatment with albiglutide was associated with a non-significant 15% lower risk of composite death from CV causes or HHF (HR 0.85, *p* = 0.113) [36]. However, albiglutide was shown to reduce the risk of incident HHF by 29% compared with placebo in a population of 20% of patients with HF at baseline [36]. The effect of albiglutide on the composite of CV death or HHF was more pronounced among patients without HF (HR 0.73 (95% CI: 0.56–0.95)). A similar pattern was observed for HHF, and albiglutide reduced the risk of first and total HHF in patients without HF at baseline by 51% and 53%, respectively [37]. During a median follow-up of 1.81 years in the AMPLITUDE-O trial, which enrolled patients with T2D and a history of CV disease or CKD, efpeglenatide reduced the risk of HHF by 39% [38], independent of concurrent SGLT2 inhibitor use (15.2% of participants) [39]. In the LEADER trial, liraglutide was associated with an 18% lower risk of the composite of HHF or CV death in patients with T2D and high CV risk, though the effect on HHF was not significant. The benefit was consistent in patients with or without a history of HF [40,41]. In other trials of GLP-1 RA in patients with T2D and high CV risk, including 8.6% to 24% having HF at baseline, treatment with exenatide [42], semaglutide [43,44], lixisenatide [45], and dulaglutide [46] did not significantly reduce the risk of HHF. In a meta-analysis of all eight trials (Table 1) involving 60,080 patients, GLP-1 RAs showed an 11% lower risk of HHF [47].

There were only three small randomized controlled trials (RCT) testing the effect of GLP-1 RAs in patients with HFrEF [46,47,48]. The LIVE trial showed that liraglutide had no effects on LVEF, quality of life, or functional class at 24 weeks in patients with chronic stable HFrEF, regardless of T2D status [48]. The FIGHT trial tested the effect of liraglutide in patients with HFrEF who were recently hospitalized for decompensation of HF. After 6 months of treatment, liraglutide had no significantly favorable effect on the global rank score (primary endpoint), death, or rehospitalizations for HF. Moreover, there was a numerically 30% higher risk for the composite outcome of death and rehospitalizations for HF overall, especially in patients with T2D [49]. In patients with HFrEF, 12 weeks of treatment with albiglutide had no significant effects on brain natriuretic peptide, LVEF, 6-minute walk test, myocardial oxygen use, or glucose consumption, though there was a slight increase in peak oxygen consumption with albiglutide 30 mg weekly. No clinical outcomes were evaluated in this trial due to the small sample size and a short follow-up period [50]. No dedicated study specifically investigating GLP-1 RAs in patients with (HFpEF) has been conducted so far. Given the substantial weight and glucose-lowering effects, the therapeutic role of GLP-1 RA may warrant further investigation in T2D obese patients with HFpEF.

### 2.3. Dual Incretin Receptor Agonists

The incretin pathway is a self-regulating feedback system connecting the brain and digestive system. It predominantly acts on postprandial glucose levels, with extra glycemic effects on fat metabolism, endovascular function, and cognitive function. Of the two main incretin hormones released with food intake, GLP-1-based therapeutics have been highly successful in obesity and diabetes management. However, glucose-dependent insulinotropic polypeptide (GIP) therapies found no clinical utility until dual incretin receptor agonists, or “twincretins”. They induce weight loss, enhance hepatic lipid metabolism, normalize systemic insulin sensitivity, and reduce or even reverse metabolic dysfunction. The US FDA approved tirzepatide as the first dual GLP-1 and GIP receptor agonist for the treatment of T2DM in 2022.

Tirzepatide, a dual incretin agonist, provided substantial, sustained, and dose-dependent reductions in BW, with a mean reduction in BW of 15%, 19.5%, and 20.9% with 5-, 10-, and 15-milligram doses, respectively, at 72 weeks in non-diabetic obese patients [51]. Tirzepatide resulted in a greater reduction in BW and glycated hemoglobin levels than semaglutide [52]. Small increases in heart rate have already been observed with the GLP-1 RA, but GIP has the potential to further increase it. However, the mean pulse rate did not differ significantly among the treatment groups [52]. A pre-specified meta-analysis included all seven RCTs with a duration of at least 26 weeks from SURPASS, a tirzepatide T2D clinical development program, suggesting that tirzepatide does not increase the risk of HHF [53]. The apparent advantage of reducing glycated hemoglobin and BW of tirzepatide over GLP-1 RA has the potential to impact the clinical management or prevention of HF. However, data on its long-term outcome are not available. The SURPASS-CVOT trial is ongoing to compare the major CV events in patients with T2D between tirzepatide and dulaglutide (NCT04255433) [54]. Another study is ongoing to evaluate the effect of tirzepatide in patients with HFpEF and obesity (NCT04847557) [55].

### 2.4. Bariatric and Metabolic Surgery

T2D is associated with obesity and multiple metabolic derangements. In addition to significant weight loss, bariatric surgery improves insulin sensitivity, β-cell function, and incretin responses leading to better lipid profiles, higher remission rates of metabolic syndrome, substantially better intestinal glucose metabolism, and brown adipose tissue metabolic activity. A recent observational study of 13, 722 obese patients with T2D showed that bariatric surgery was associated with a 62% reduction in the risk of incident HF (HR, 0.38 (95% CI 0.30–0.49)) in comparison with nonsurgical management [56]. Another observational study of 5321 T2D patients receiving Roux-en-Y gastric bypass (RYGB) surgery and 5321 matched T2D controls reported a 73% lower risk for HF (HR, 0.27 (95% CI: 0.19–0.38)). In patients with preexisting HF, the risk reduction was even higher (HR, 0.23 (95% CI: 0.12–0.43)) [57]. Another observational study, including 1362 T2D patients receiving RYGB, 693 T2D patients receiving sleeve gastrectomy, and 11435 matched nonsurgical T2D controls, found RYGB was associated with a 68% reduced risk (HR, 0.32 (95% CI: 0.23–0.44)) and SG was associated with a 60% reduced risk of incident HF (HR: 0.40 (95%: 0.25–0.66)) compared with controls [58]. Although there is currently no RCT for the effect of bariatric or metabolic surgery on HF in T2D patients, large observational studies provide strong support for the substantial benefit of bariatric or metabolic surgery on HF in T2D patients [56,57,58].

### 2.5. Bromocriptine Mesylate

#### 2.5.1. Cardiovascular Benefits in Diabetic Patients

Bromocriptine-QR, a quick-release formulation of bromocriptine mesylate, was approved by the US FDA for T2D in 2009. It is a fast-acting sympatholytic dopamine D2 receptor agonist that was originally used as an inhibitor of prolactin for the treatment of hyperprolactinemia. In a number of clinical trials, bromocriptine reduced fasting and postprandial glucose levels, corrected dyslipidemia, and improved cardiovascular outcomes [56,57,58,59,60,61,62,63].

In a large phase III double-blinded RCT recruiting 3070 participants, bromocriptine treatment for 52 weeks resulted in a 39% reduction in CV death-inclusive composite CV endpoints, including myocardial infarction, stroke, hospitalized angina, HHF, coronary revascularization, and CV death (HR: 0.61 (95% CI: 0.38–0.97)). Of note, there was a non-significant 28% reduced risk in HHF (HR, 0.77 (95% CI: 0.27–2.16)), probably owing to the relatively limited HHF events [62]. These data demonstrated a substantial cardiovascular benefit of bromocriptine in patients with diabetes.

#### 2.5.2. Therapeutic Efficacy in Peripartum Cardiomyopathy

Peripartum cardiomyopathy, also known as postpartum cardiomyopathy, is responsible for the majority of HF in pregnant women from the last month of pregnancy to five months after delivery. A recent meta-analysis of eight studies involving 593 patients reported that bromocriptine treatment increased LVEF (53.3% vs. 41.8%, *p* < 0.001) and prolonged survival (91.6% vs. 83.9%, *p* = 0.02) [63]. Based on these findings, a “BOARD” regimen, including bromocriptine, oral HF drugs, anticoagulants, vaso-relaxants, and diuretics, was proposed for treating peripartum cardiomyopathy [64].

### 2.6. Imeglimin

Imeglimin was approved in 2021 as a new anti-diabetic agent. Imeglimin has been shown to exert pleiotropic effects, including the augmented glucose-stimulated insulin secretion of pancreatic β-cells, improved insulin sensitivity in muscle, and, most importantly, reduced hepatic gluconeogenesis [65].

There is currently no RCT in regard to the effect of imeglimin on HF. However, several preclinical studies demonstrated the therapeutic effect of imeglimin on HF in diabetic rodents [66,67] and the prevention of endothelial cell death [68].

### 2.7. Metformin

Metformin is a biguanide derivative that reduces glucose production from the liver, decreases intestinal absorption, and increases insulin sensitivity. Metformin is considered weight neutral and decreases both basal and postprandial blood glucose levels. Metformin was previously contraindicated in patients with HF. However, abundant observational studies supported a beneficial effect of metformin on HF. A meta-analysis of 11 observational studies involving 35,950 T2D patients with HF reported a 22% reduction in total mortality (HR, 0.78, (95%: 0.71–0.87)) in those receiving metformin [69]. Another meta-analysis of nine observational studies pooling 34,504 T2D patients with HF receiving metformin versus controls (mostly sulfonylurea) reported a 20% reduction in mortality (HR: 0.80, (95% CI: 0.74–0.87)) and a 7% reduction in all-cause hospitalization (HR, 0.93; (95% CI: 0.89–0.98)) [67]. Of note, there was a non-significant 9% reduction in mortality in those with severe left ventricular dysfunction (HR, 0.91 (95% CI: 0.72–1.14)) [70]. A recent propensity score-matched observational study in 847 T2D patients with advanced HFrEF reported markedly lower B-type natriuretic peptide (BNP) levels, improved LVEF, and better event-free survival in metformin users than in controls irrespective of glycemic control [71]. However, these results should be interpreted cautiously, since metformin is often avoided in patients with multiple comorbidities, including HF and their comparators, such as sulfonylurea or SLT2 inhibitors, which may alter the estimated risk of HF.

In contrast, meta-analyses of RCTs reported no benefit of metformin on HF in diabetic patients [72,73]. In a post-hoc analysis of the patients in the Saxagliptin and Cardiovascular Outcomes in Patients with Type 2 Diabetes Mellitus trial (SAVOR-TIMI 53) involving 12,156 patients with T2D with or without HF or renal dysfunction, metformin use was associated with no changes in incident HHF (HR, 0.97 (95%: 0.77–1.23), *p* = 0.80) after adjustment for clinical variables and biomarkers [74].

However, a double-blinded placebo-controlled RCT in 36 insulin-resistant patients with HFrEF showed that metformin treatment for 3 months improved myocardial work metabolic index, myocardial external efficiency, myocardial oxygen consumption, and positron emission tomography (PET)-derived stroke work assessed by 11C-acetate PET [75]. Therefore, the results of the ongoing DANHEART trial (NCT03514108) evaluating the effect of metformin in patients with HFrEF and T2D [76] and the Investigation of Metformin in Pre-diabetes on Atherosclerotic Cardiovascular Outcomes (VA-IMPACT) trial (NCT02915198) investigating the effect of metformin on atherosclerotic CV outcomes in 7410 prediabetic participants are eagerly awaited [77].

### 2.8. Insulin

In the ORIGIN RCT, involving 12,537 patients with a median follow-up of 6.2 years, insulin glargine treatment was associated with no change in HHF (HR, 0.90 (95% CI: 0.77–1.05), *p* = 0.15) [78]. In the Bypass Angioplasty Revascularization Investigation 2 Diabetes (BARI 2D) trial, recruiting 2368 patients with T2D and coronary artery disease, the incident HF did not differ significantly between patients receiving insulin-sensitization therapy (metformin or thiazolidinediones) (19.4%) and those receiving insulin-provision therapy (insulin or sulfonylurea) (16.6%, *p* = 0.09) [79]. However, this result should be interpreted cautiously, since thiazolidinediones are associated with an increased risk of HF, and metformin might be protective against HF.

Around 30% of patients comorbid with HF and DM received insulin therapy, but no specific RCTs of insulin on clinical outcomes have been conducted. In RCTs focusing on DM, such as the DECLARE-TIMI 58, around 40% of patients were prescribed insulin. On the other hand, in RCTs focused on HF, such as the DAPA-HF, 27% of patients were given insulin. However, insulin was associated with a higher risk of all-cause mortality and HHF in a recent post-hoc analysis of three RCTs [80].

In the administrative registry, insulin prescription was associated with a higher risk of all-cause death (odds ratio (OR) 2.02, 95% CI 1.87–2.19) and rehospitalization for HF (OR 1.42, 95% CI 1.32–1.53) [81]. Moreover, even after traditional risk factors adjustments, insulin-resistant states such as T2D mellitus and obesity increase the risk of HF. The alteration of proximal insulin-signaling pathways may contribute to adverse left ventricular remodeling and mitochondrial dysfunction. The changes in distal elements of insulin signaling pathways such as forkhead box O transcriptional signaling or glucose transport may also impair cardiac structure, metabolism, and function [82].

As insulin was often used in patients of advanced DM stage, it was reasonably associated with poor outcomes in HF, which should be investigated further with controlled trials.

### 2.9. Sulfonylurea

Sulfonylureas are a class of anti-diabetic compounds widely used in the treatment of T2D by increasing insulin release from the beta-cells in the pancreas. They were used in 20–40% of patients with DM and HF. In the UKPDS 33 trial comparing intensive glycemic control using sulfonylurea or insulin (2729 patients) versus conventional therapy (1138 patients), the result showed no difference in incident HF in 3867 newly diagnosed T2D patients (HR, 0.91 (95% CI, 0.54–1.52)) [83].

However, sulfonylurea was associated with an increased risk of death in patients with T2D and HF in observational studies. In a retrospective cohort study in Canada, comparing sulfonylureas to other oral anti-diabetic agents as an add-on therapy to metformin, patients prescribed sulfonylurea had a higher risk of all-cause mortality (HR 1.44, *p* = 0.005) and major hypoglycemic episodes (HR 2.78, *p* < 0.001) [84]. In a registry of 5852 patients hospitalized for HF with comorbid T2D between 2006 and 2014, sulfonylurea initiation was associated with an increased risk of mortality (HR: 1.24; 95%, *p* = 0.045) and HHF (HR: 1.22, *p* = 0.050), regardless of ejection fraction (all P for interaction >0.11) [85]. In a retrospective cohort study in T2D patients with impaired renal function, 24,685 metformin users had a lower rate of HHF than 24,805 sulfonylurea users (HR, 0.85 (95% CI, 0.78–0.93)) [86].

### 2.10. Alpha-Glucosidase Inhibitors

Alpha-glucosidase inhibitors (AGI) delay the digestion and absorption of carbohydrates by competitively inhibiting the enzymes that cleave oligosaccharides to monosaccharides in the brush border of the small intestine. Acarbose, which is the main product of AGI, has been shown to reduce postprandial hyperglycemia and decrease BW, and is associated with a lower risk of hypoglycemia. In a meta-analysis of seven RCTs, treatment with acarbose for 52 weeks did not significantly reduce the risk of HF in T2D patients [87]. However, there were only 7 events in the acarbose group versus 10 in the placebo group of more than 2000 patients. Therefore, no firm conclusion can be drawn from the meta-analysis data. The Acarbose Cardiovascular Evaluation (ACE) trial in 6522 Chinese patients with coronary heart disease and impaired glucose tolerance also showed no significant difference in HHF between the acarbose-treated group and the placebo group (HR, 089 (95% CI: 0.63–1.24)) [88].

### 2.11. Thiazolidinediones (TZD)

Thiazolidinediones are potent synthetic ligands of peroxisome proliferator-activated receptors γ (PPARγ). Pioglitazone has been shown to prevent atherosclerotic events in several RCTs [89,90,91]. However, as a class effect, thiazolidinediones causes fluid retention through the upregulation of the epithelial sodium channel (ENaC) in the collecting ducts [92] and other transporters in the proximal tubule such as sodium bicarbonate co-transporter, sodium–proton exchangers, and water channels. The expanded fluids increase the risk of leg edema and HF, especially in combination with insulin therapy. In the Diabetes Reduction Assessment with Ramipril and Rosiglitazone Medication (DREAM) study, rosiglitazone markedly increased the risk of HHF (HR, 7.04 (95% CI, 1.60–31.0)) [93]. In the PROactive trial, pioglitazone significantly increased HHF (6% vs. 4%, *p* = 0.007) and HF not needing hospitalization (5% vs. 3%, *p* = 0.003) [90]. Another RCT involving 224 T2D patients with NYHA class I and II showed that rosiglitazone increased CV hospitalization (19.1% vs. 13.2%), worsening of HF (6.3% vs. 3.5%), worsening of edema (25.5% vs. 8.8%), and increased HF medication (32.7% vs. 17.5%) [94]. A meta-analysis of RCTs, including 20191 participants compared with the controls, revealed an increased risk of HF (RR 1.72, (95% CI, 1.21–2.42), *p* = 0.002) [95]. Therefore, the American Diabetes Association and the American Heart Association recommended that thiazolidinediones should be avoided in patients with NYHA class III or IV HF [96].

### 2.12. Dipeptidyl Peptidase-4 (DPP-4) Inhibitors

DPP-4 is an enzyme that breaks down incretin hormones, mainly GLP-1 (glucagon-like peptide-1) and GIP (gastric inhibitory peptide). These incretins are released within a few minutes of food intake, increasing insulin secretion and decreasing glucagon secretion to maintain glucose homeostasis, and DPP-4 degrades these hormones immediately. DPP-4 inhibitors, also known as gliptins, increase the levels of GLP-1 and GIP, thereby reducing postprandial and fasting hyperglycemia. Saxagliptin and possibly alogliptin were associated with HHF in the Saxagliptin Assessment of Vascular Outcomes Recorded in Patients with Diabetes Mellitus—Thrombolysis in Myocardial Infarction 53 (SAVOR-TIMI53 trial) (HR, 1.27 (99% CI: 1.07–4.51)) [97]. In the Examination of Cardiovascular Outcomes with Alogliptin versus Standard of Care (EXAMINE) trial, alogliptin was associated with a non-significant 19% increase in the risk of HHF (HR, 1.19 (95% CI: 0.90–1.58)). The risk differed among patients with prior history of HF (HR, 1.00 (95% CI: 0.71–1.42)) and those without prior history of HF (HR, 1.76 (95% CI: 1.07–2.90)) [98]. Additionally, vildagliptin has adverse effects on cardiac remodeling and a higher risk of CV hospitalization and death in the Vildagliptin in Ventricular Dysfunction Diabetes (VIVIDD) trial in patients with DM and left ventricular systolic dysfunction [99]. The underlying mechanism includes the blockade of sodium reabsorption in the renal tubules [100], increases in myocardial cAMP via increases in GLP-1 [101], and increased circulating stromal cell-derived factor 1 (SDF-1), substance P, and neuropeptide Y (NPY), which are substrates of DPP4. These peptides have been shown to cause myocardial fibrosis, activate sympathetic tone, and accelerate cardiomyocyte apoptosis [102,103,104,105,106]. However, in the Trial Evaluating Cardiovascular Outcome with Sitagliptin (TECOS) RCT, sitagliptin was not associated with an increased risk of HHF (HR, 1.0 (95% CI: 0.83–1.20)) [107]. In the Cardiovascular Safety and Renal Microvascular Outcome Study with Linagliptin (CARMELINA) RCT, linagliptin was not associated with an increased risk of HHF (HR, 0.9 (95% CI: 0.74–1.08)) compared with placebo [108]. In the Cardiovascular Outcome Trial of Linagliptin versus Glimepiride in Type 2 Diabetes (CAROLINA) RCT, linagliptin use was also not associated with an increased risk of HHF compared with glimepiride (HR: 1.21 (95% CI: 0.92–1.59)) [109], suggesting that the harmful effect of DPP-4 inhibitors on HF are not class effects.

**Table 1:** Randomized clinical trials of the effect of anti-diabetic therapy on heart failure or hospitalized heart failure.

Therapy	Condition	Trial	Reference	Agent	No.	Follow-Up (Years)	Mean Age	Women (%)	DM (%)	HF (%)	CV Death or HHF (HR)	HHF (HR)
SGLT2i	DM	EMPAREG OUTCOME	[5,8]	Empagliflozin	7020	3.1	63	29	100	10	0.66 *	0.65 *
		CANVAS	[6,9]	Canagliflozin	10,420	2.4	63	36	100	14	0.78 *	0.67 *
		DECLARE-TIMI 58	[7,10]	Dapagliflozin	17,160	4.2	64	37	100	10	0.83 *	0.73 *
		VERTIS CV	[11,12]	Ertugliflozin	8246	3.5	64	30	100	24	0.88	0.70 *
	CKD	CREDENCE	[15,18]	Canagliflozin	4041	2.6	63	34	100	15	0.69 *	0.61 *
		SCORED	[16]	Sotagliflozin	10,508	1.3	69	45	100	31	0.77 *	0.67 *^#^
		DAPA-CKD	[17,19]	Dapagliflozin	4304	2.4	62	33	68	11	0.71 *	0.51 *
		EMPA-KIDNEY	[20]	Empagliflozin	6609	2	64	33	46	10	0.84	0.8
	HF	DAPA-HF	[21,22]	Dapagliflozin	4744	1.5	66	23	45	100	0.75 *	0.70 *
		EMPEROR-Reduced	[23]	Empagliflozin	3730	1.3	67	24	50	100	0.75 *	0.69 *
		EMPEROR-Preserved	[24]	Empagliflozin	5988	2.2	72	45	49	100	0.79 *	0.71 *
		DELIVER	[25]	Dapagliflozin	6263	2.3	72	44	45	100	0.80 *	0.77 *
		SOLOIST-WHF	[26]	Sotagliflozin	1222	0.75	70	34	100	100	0.71 *	0.64 *^#^
GLP-1 RA	DM	Harmony Outcomes	[28,29]	Albiglutide	9463	1.6	64	31	100	20	0.85	0.71 *
		AMPLITUDE-O	[30]	Efpeglenatide	4076	1.8	65	33	100	18	-	0.61 *
		LEADER	[32,33]	Liraglutide	9340	3.8	64	36	100	18	0.82 *	0.87
		EXSCEL	[34]	Exenatide	14,752	3.2	62	38	100	16	-	0.94
		SUSTAIN 6	[35]	Semaglutide	3297	2.1	65	39	100	24	-	1.11
		PIONEER 6	[36]	Semaglutide (oral)	3183	1.3	66	32	100	12	-	0.86
		ELIXA	[37]	Lixisenatide	6068	2.1	60	31	100	22	-	0.96
		REWIND	[38]	Dulaglutide	9901	5.4	66	46	100	9	-	0.93 ^&^
Bromocriptine	DM		[54]	Bromocriptine	3070	1	60.1	57	100	-	-	0.77
Metformin	DM	SAVOR-TIMI 53 (post hoc)	[66]	Metformin	12,156	2.1	62.4	33.2	100	13.18	-	0.97
Insulin		ORIGIN	[70]	Glargine	12,537	6.2	63.5	34.8	100	-	-	0.90
Insulin or SU	DM + CAD	BARI 2D	[71]	Insulin or SU	2368	5	62.4	29.6	100	21.3	-	0.86
Insulin or SU	DM	UKPDS 33	[75]	Insulin or SU	3867	10	54	38.9	100	-	-	0.91
α-glucosidase inhibitor	Prediabetes+ CAD	ACE	[80]	Acarbose	6552	5	64.3	27	0	0		0.89
TZD	Prediabetes	DREAM	[85]	Rosiglitazone	5269	3	54.7	59.2	0	0		7.04 *
	DM + HF		[86]	Rosiglitazone	224	1	62.1	18.32	100	100		1.8 ^@^
		PROactive	[82]	Pioglitazone	5238	2.85	61.7	33.5	100		0	1.49 *
DDP-4 inhibitor	DM	TECOS	[99]	Sitagliptin	14,671	3	65.5	29.3	100	18		1.00
		SAVORP-TIMI53	[89]	Saxagliptin	16,492	2.1	65.1	33.1	100	12.7		1.27 *
		EXAMINE	[90]	Alogliptin	5380	1.46	61	32.1	100	28.5		1.07 *
		CARMELINA	[100]	Linagliptin	6979	2.2	65.85	35.7	100	26.8		0.90
		CAROLINA	[101]	Linagliptin	6033	6.3	64	39.9	100	4.6		1.21
Life Style Intervention	DM + Overweight/Obesity	Look AHEAD	[103]	Moderate physical activity and calorie restriction	5109	12.4	59.1	59.43	100	-		0.96

* *p* < 0.05. ^#^ Total no. of HHF and urgent visits for HF. ^&^ HHF or urgent HF visit. ^@^ Worsening of HF.

### 2.13. Pramlintide

Pramlintide is a synthetic analog of the pancreatic peptide amylin. Both amylin and pramlintide have similar effects on lowering postprandial glucose and glucagon, delaying gastric emptying, and promoting satiety via hypothalamic receptors. A meta-analysis of five RCTs, including 1434 pramlintide-treated patients and 582 controls, reported a neutral effect of major adverse CV events [110]. However, there is no observational study or RCT addressing the effect of pramlintide on HF.

### 2.14. Lifestyle Intervention

Lifestyle interventions, including diet control, exercise, and weight loss, are essential components of T2D treatment. However, in the Action for Health in Diabetes (Look AHEAD trial) RCT, overweight or obese T2D patients receiving intensive lifestyle intervention, including an average 7% weight loss, a calorie-restricted diet (1200–1800 kcal/day), and moderate-intensity physical exercise did not change the risk of incident HF (HR, 0.96 (95% CI: 0.75–1.23)) [111].

## 3. Anti-Diabetic Drugs and Heart Failure: Recent Progress of Molecular Mechanism

### 3.1. SGLT2 Inhibitors

**Figure 1:**
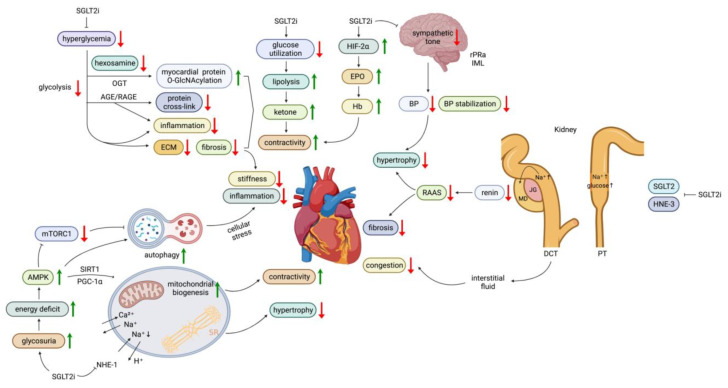
Molecular mechanism of the effect of sodium-glucose co-transporter-2 inhibitors on heart failure. SGLT2i: sodium-glucose co-transporter-2 inhibitors; OGT: O-linked N-acetylglucosaminyltransferase; O-GlcNAcylation: O-linked acetylglucosaminylation; AMPK: the 5′ adenosine monophosphate-activated protein kinase-activated protein kinase; PGC1α: the peroxisome proliferator-activated receptor gamma coactivator 1-alpha; mTOR: the mammalian target of rapamycin; RAAS: renin-angiotensin-aldosterone; HIF2α: hypoxia-inducible factor 2α; NHE3: the apical Na+/H+ exchanger isoform 3; EPO: erythropoietin; rRPa: the rostral raphe pallidus nucleus; IML: intermediolateral nucleus of spinal cord; AGE: advanced glycation end products; RAGE: receptor for advanced glycation end products; PKC: the protein kinase C; NHE1: Na+-H+ exchanger isoform 1; NLRP3: the NLR family, pyrin domain-containing 3; SR: sarcoplasmic reticulum; MD: macular densa; JG: juxtaglomerular cells; DCT: distal convoluted tubules; PT: proximal tubules.

The mechanisms are summarized in Figure 1.

#### 3.1.1. Effects of SGLT1 Inhibition

**Figure 2:**
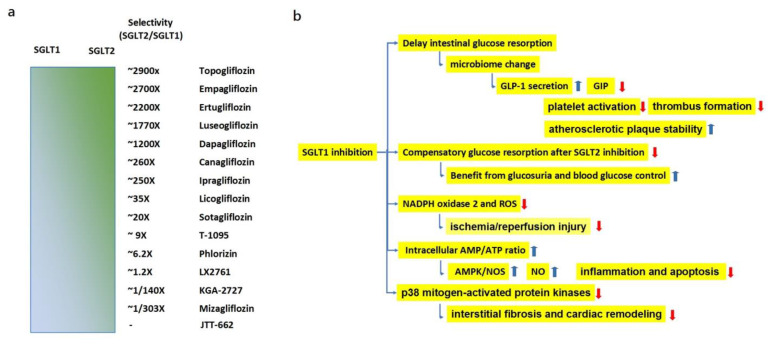
(**a**) Selectivity comparison of SGLT inhibitors. (**b**) Mechanism by which SGLT1 inhibition improves cardiovascular outcome. GLP-1: glucagon-like peptide receptor; GIP: glucose-dependent insulinotropic polypeptide; NADPH: nicotinamide adenine dinucleotide phosphate; ROS: reactive oxygen species; AMP: the 5′ adenosine monophosphate; ATP: the 5′ adenosine triphosphate; AMPK: the 5′ adenosine monophosphate-activated protein kinase-activated protein kinase; NOS: nitric oxide synthase; NO: nitric oxide.

Based on the molecular structure of phlorizin, a natural dual SGLT1/2 inhibitor, several SGLT2, SGLT1, and dual SGLT inhibitors have been developed. The sodium glucose co-transporter (SGLT) inhibitors have proven effective in preventing HF and HHF in patients with T2D independent of LVEF [112]. However, there are major differences in the proportional inhibition of SGLT1 and SGLT2 among them. Particularly, the selectivity for SGLT2 compared with SGLT1 varies from 1/303 times to 2900 times (Figure 2a) [113]. Of the SGLTs, which comprise at least six different isoforms in humans, SGLT1 and SGLT2, frequently investigated, play key roles in the transportation of sodium and glucose across the brush border membrane of intestine and renal tubules. Physiological roles of other SGLTs remain unknown.

SGLT1 is often upregulated when SGLT2 is knockout or inhibited by treatment. Therefore, the combination therapy of an SGLT1 inhibitor and an SGLT2 inhibitor or the monotherapy with a dual inhibitor is expected to block both pathways and induce significant glucosuria and glycemic control than either an SGLT1 or SGLT2 inhibitor alone [114].

SGLT1 inhibition also reduced glucose and hemoglobin A1C levels in patients with an eGFR < 30 mL/(min·1.72 m^2^) to a similar degree as in those with an eGFR ≥ 30 mL/(min·1.72 m^2^) [22]. Thus, a relative increase in SGLT1 inhibition may be superior in lowering hemoglobin A1C levels and preventing downstream microvascular complications of T2D in patients with CKD. Moreover, SGLT1 is found in the capillaries of the heart, brain, and skeletal muscle, and it has been shown to be upregulated in patients with diabetic cardiomyopathy [22].

The SGLT1 inhibitors delay the absorption of monosaccharides, increase the glucose delivery to the distal intestines, and decrease intestinal pH. As a result, there is a change in the intestinal microbiome, an increase in short-chain fatty acids, an increase in the secretion of GLP-1, and a decrease in GIP [115]. The increase of plasma GLP-1 after meals was also observed in healthy humans treated with SGLT1 inhibitor [116] and patients with T2D treated with SGLT1/2 inhibitor [117]. The possible mechanism of GLP-1 release is the fermentation of glucose to short-chain fatty acids at the distal parts of the small intestine.

The dual SGLT1/2 inhibitor sotagliflozin reduced CV mortality and HHF in patients with HF, both in HFrEF as well as HFpEF, prevented the development of HF in patients with T2D and CKD in the SCORED [22], and SOLOIST [32] trials, similar to the benefits by other SGLT2 inhibitors. Nevertheless, it decreases the incidence of non-fatal and fatal stroke by 30% and myocardial infarction by 30% [22].

Sotagliflozin might be superior to highly selective SGLT2 inhibitors in terms of HF outcome [118]. The possible explanation is that SGLT1 receptors are specifically expressed in the human myocardium [119,120]. Myocardial SGLT1 was upregulated in patients with end-stage HF [121], and is significantly correlated with cardiac remodeling and systolic function [121]. The benefit of atherosclerotic diseases is also postulated to be associated with the effect of SGLT1i on GLP-1, resulting in a decrease in platelet activation, reduction of thrombus formation, and an increase in atherosclerotic plaque stability [22].

Being associated with an increase in nicotinamide adenine dinucleotide phosphate (NADPH) oxidase 2 and reactive oxygen species, SGLT1 knockdown attenuates ischemia/reperfusion injury [122]. Canagliflozin, with relatively lower SGLT2/SGLT1 selectivity, had been shown to suppress myocardial NADPH oxidase activity and improves NOS coupling via SGLT1/AMPK/Rac1 signaling, leading to global anti-inflammatory and anti-apoptotic effects in the human myocardium [123].

Canagliflozin increases the intracellular AMP/ATP ratio by inhibiting SGLT1, then activates AMPK/nitric oxide synthase (NOS) signals and increases nitric oxide (NO), which inhibits pro-inflammatory signals [123]. The activation of AMPK also inhibits the activation of Rac1 and the membrane translocation of p47phox and Rac1, thereby suppressing Nox activity and O2 generation, decreasing inflammation and apoptosis pathways. However, the SGLT2-specific inhibitor empagliflozin, which has almost no affinity to SGLT1, did not affect myocardial Nox activity, O2 production, or NOS coupling status [123]. Considering that SGLT2 is not expressed in the heart, this effect might be linked to SGLT1 inhibition.

SGLT1 may be a new therapeutic target to alleviate ischemia and reperfusion injury. The overexpression of SGLT1 increases the phosphorylation of p38 mitogen-activated protein kinases and facilitates fibroblast proliferation and collagen synthesis, which in turn induces interstitial fibrosis and cardiac remodeling [124].

SGLT1 suppression is also linked to reductions in ventricular hypertrophy and myocardial fibrosis. These effects could further contribute to a reduction in heart failure, especially heart failure with preserved left ventricular function, where both left ventricular hypertrophy and fibrosis are thought to play an important role [125]. SGLT1 expression increased in patients with diabetes cardiomyopathy and in streptozotocin diabetes rats; inhibiting SGLT1 attenuates apoptosis and inhibits the development of diabetes cardiomyopathy. Therefore, SGLT1 inhibition might reduce the occurrence of heart failure independent of SGLT2 inhibition. Heterozygosity for the missense variants in SLC5A1 (solute carrier family 5 member 1), which cause decreased SGLT1 function, was associated with decreased incidence of heart failure and death, as well as T2D [126].

A recent study reported that chronic cardiac overexpression of SGLT1 in mice led to pathological cardiac hypertrophy and left ventricular failure, and cardiac knockdown of SGLT1 attenuated the disease phenotype [127]. In contrast, a recent study also reported that dual SGLT1/SGLT2 inhibitors exacerbated cardiac dysfunction after experimental myocardial infarction in rats [128].

Some highly SGLT1-selective compounds are under investigation. The selective SGLT1 inhibitor KGA-2727 has been revealed to have a protective effect on ischemic cardiomyopathy post-myocardial infarction (MI) [129]. In addition, experiments have proved that mizagliflozin significantly improves cardiac function with concentration dependence in diabetic rats [124]. LX2761 is another SGLT 1 inhibitor restricted to the intestinal lumen after oral administration. Treating mice with LX2761 and the dipeptidyl-peptidase 4 inhibitor sitagliptin synergistically increased active GLP-1 levels [130].

Although SGLT1 has been found in several other organs, the lack of a clear understanding of the function of SGLT1 in these tissues, the effect of SGT1 inhibition on the microbiome, and the alteration of SGLT1 expression in various tissues on comorbidities, such as HF and T2D, suggest the need for further investigation of SGLT1 and SGLT1/2 inhibitors.

#### 3.1.2. Effects of SGLT2 Inhibitors on Energy Utilization

Under normal conditions, cardiomyocytes generate ATP mainly from fatty acid oxidation, with a minor contribution from glucose. Myocardial ketone body utilization is suppressed in diabetes, probably due to impaired mitochondrial function where the ketone body is converted into acetyl-CoA [131]. The loss of ketone utilization capability impairs myocardial function. The cardiomyocyte-specific knockout of succinyl-CoA:3 ketoacid-CoA transferase, the key enzyme for the terminal step of ketone body oxidation, leads to cardiomegaly [132]. SGLT2 inhibition induces lipolysis due to reduced carbohydrate usage and an altered glucagon/insulin ratio, leading to increased ketogenesis. Ketones may provide a more efficient energy source for patients with a failing heart [133].

Autophagy is a lysosome-dependent self-degradative process that recycles damaged organelles to provide an energy source during nutrient deficiency. In diabetes, an over-nutritional condition, autophagy is suppressed through the activation of the mammalian target of rapamycin (mTOR) pathway, a nutrient-sensing pathway [134].

In contrast, the 5′ adenosine monophosphate-activated protein kinase-activated protein kinase (AMPK) is an energy deficiency-sensing pathway and is suppressed in diabetes [135]. The activation of the AMPK pathway suppresses the mTOR pathway and activates the peroxisome proliferator-activated receptor gamma coactivator 1-alpha (PGC1α) transcriptional factor, a main regulator of mitochondrial function [136].

The sirtuin pathway is a nicotinamide adenine dinucleotide (NAD)-responsive deacetylase that also senses cellular energy deficiency. The activation of the sirtuin pathway promotes mitochondrial biogenesis in diabetes [137].

In animal models, empagliflozin reduces the post MI mortality rate by modification of cardiac energy metabolism and antioxidant proteins [138]. It alleviates atrial remodeling and improves mitochondrial function in diabetic rats by activating the PGC1α pathway [139]. Empagliflozin also rescues myocardial microvascular injury via the AMPK pathway in diabetic mice [140].

SGLT2 inhibitors induce calorie loss via glucosuria in diabetes, which activates the AMPK and sirtuin pathway and increases autophagy through mTORC1 suppression. Empagliflozin enhances autophagy in the myocardium via the activation of the AMPK pathway and attenuates cardiac inflammation in diabetic rats [141]. Empagliflozin improved mitochondrial oxidative phosphorylation, enhanced mitochondrial biogenesis, increased autophagy, decreased cardiac apoptosis, and reduced reactive oxygen species (ROS) in a transverse aortic constriction-induced pressure-overload heart failure mouse model, leading to attenuated transverse aortic constriction-induced cardiac dysfunction and ventricular remodeling without changes in glucose or weight [142].

The hypoxia-inducible factor (HIF) pathway responds to hypoxia. The activation of HIF-1α decreases oxygen consumption and reactive oxygen species (ROS), while HIF-2α enhances erythropoietin (EPO) synthesis. Diabetes is characterized by the activation of HIF-1α and the suppression of HIF-2α. Recent evidence demonstrates that SGLT2 inhibitors selectively activate HIF-2α [143]. Dapagliflozin has been shown to attenuate cardiac fibrosis and inflammation by activating the HIF-2α-signaling pathway in arrhythmogenic cardiomyopathy [144].

#### 3.1.3. Effects of SGLT2 Inhibitors on Renin-Angiotensin-Aldosterone (RAAS) Activation

SGLT2 is distributed mainly in proximal renal tubules and reabsorbs 90% of filtered glucose and 60–70% of sodium chloride and water [145]. The expression of SGLT2 substantially increases in animal models of type 1 diabetes (T1D) and T2D [146]. The increased SGLT2 expression increases the proximal tubular reabsorption of sodium and glucose and thus reduces sodium concentration in renal tubules, which disrupts the “tubular–glomerular feedback” and activates the RAAS axis.

SGLT2 inhibitor treatment attenuates proximal tubular glucose and sodium reabsorption and maintains macula densa exposure to high sodium concentrations, resulting in decreased renin secretion, reduced activation of the RAAS axis, and lowered glomerular filtration [147].

In addition, the apical Na+/H+ exchanger isoform 3 (NHE3), which actively reabsorbs ~30% of sodium in proximal tubules, contributes to tubular sodium hyper-reabsorption in diabetes-associated hyperfiltration. Hyperglycemia or the pharmacological inhibition of SGLTs inhibits this exchanger [148]. In diabetic mice, chronic SGLT2 inhibitor treatment decreases sodium uptake by NHE3 [149]. In diabetic rats, empagliflozin decreased the tubular expression of NHE3 and the epithelial sodium channels [150], which reduced sodium uptake by NHE3, thus maintaining the urine sodium concentration. The maintained urine sodium concentration in distal convoluted tubules reduced the response of the macula densa and the release of renin from adjacent juxtaglomerular cells, leading to alleviated RAAS activation.

#### 3.1.4. Effects of SGLT2 Inhibitors on Sodium and Fluid Dynamics

Patients with diabetes have a higher incidence of asymptomatic HF, in part due to excessive sodium intake and decreased sodium excretion as diabetic nephropathy progresses. In a human study, SGLT2 inhibitors modulated diuresis and weight loss by blocking sodium and glucose reabsorption [151]. The natriuretic effect of SGLT2 reduces the sodium burden in diabetic patients. Dapagliflozin produces a 200% greater loss in interstitial fluid volume, while the relative reduction of interstitial fluid volume with bumetanide is only 78%. Thus, by reducing the interstitial fluid volume to a greater extent than blood volume, SGLT2 inhibitors may prevent congestion without hindering arterial perfusion [152].

The reduced erythropoietin (EPO)-producing ability in patients with diabetes has been reversed after treatment with SGLT2 inhibitors [153]. The increased hematocrit augments oxygen delivery, providing an additional cardioprotective effect in patients with congestive heart failure.

#### 3.1.5. Effects of SGLT2 Inhibitors on the Sympathetic Nervous System

The sympathetic nervous system is activated in patients with HF, which further deters cardiac function. Unlike traditional diuretics, a recent study suggested that the SGLT2 inhibitor-mediated natriuresis did not directly activate sympathetic tone [154]. A meta-analysis revealed that SGLT2 inhibitors reduced BP without a compensatory increase in heart rate [155]. These findings suggest that sympathetic modulation may be one of the key players in the cardioprotective effects of SGLT2 inhibitor treatment.

Sympathetic nerves innervate the proximal tubules of the kidney. Therefore, it has been proposed that the effect of SGLT2 inhibition of sympathetic tone is secondary to a reduction in renal afferent sympathetic activation. However, evidence indicates that SGLT2 inhibitors directly act on the central nervous system. In addition, they promote parasympathetic nervous activity, which decreases BP and heart rate [156]. SGLT2 inhibitors have beneficial effects on morning BP, and nocturnal BP in diabetic rats [157]. The normalized diurnal BP abnormality, which commonly occurs in patients with diabetes, may prevent or treat HF in diabetic patients.

Dapagliflozin has been shown to reduce norepinephrine turnover and decrease the expression of tyrosine hydroxylase, the rate-limiting enzyme of catecholamine biosynthesis, production in brown adipose tissue through decreased sympathetic input from the rostral raphe pallidus nucleus (rRPa) [158]. Another study showed that dapagliflozin likely reduced BP by modulating the sympathetic signals from the rostral ventrolateral medulla (RVLM) to regulate the sympathetic impulses to the intermediolateral nucleus of the spinal cord (IML) with sympathetic preganglionic neurons [159].

SGLT2 inhibitors also restored impaired cardiac autonomic neuropathy and stabilized BP fluctuations in diabetic rats [135] and diabetic patients [160]. Taken together, the normalized autonomic disturbance by SGLT2 inhibitors may benefit myocardial function in diabetic patients.

#### 3.1.6. Anti-Inflammatory and Anti-Fibrotic Effects of SGLT2 Inhibitors

The hyperglycemia flux-induced generation of glycolysis intermediates provokes the advanced glycation end products (AGE), the protein kinase C (PKC) pathway, and the hexosamine pathways. AGEs are pro-inflammatory and pro-oxidant substances that promote collagen cross-linking, leading to myocardial stiffness [161]. SGLT2 inhibitor treatment reduces cardiac oxidative stress by reducing AGE products within the myocardium or aorta [162].

O-GlcNAc transferase (O-linked N-acetylglucosaminyltransferase) is an enzyme for the post-translational modification of residues of serine and threonine residues of proteins, including many important proteins involved in myocardial function such as phospholamban, calmodulin kinase II, and troponin I. The hyperglycemia-induced hexosamine pathway supplies the N-acetylglucosamine moiety (O-GlcNAc) O-linked to these proteins by OGT. Thus, the chronic activation of the hexosamine pathway in diabetic hearts affects Ca^2+^ handling, contractile properties, and ventricular hypertrophy [163]. The SGLT2 inhibitor dapagliflozin has recently been shown to lower cardiac hexosamine flux and reduce the O-GlcNAcylated protein levels, thus preventing HF in diabetic mice [164].

The PKC pathway is involved in the enhancement of oxidative stress, cytokine and growth factor actions, leukocyte adhesions, extracellular matrix (ECM) accumulation, and the regulation of cardiomyocyte proliferation [165,166]. SGLT2 inhibitors inhibit complex I of the mitochondrial respiratory chain, which subsequently increases adenosine monophosphate (AMP) and ADP content [167]. Elevated AMP/ADP binds to the g subunit of AMPK, activating its phosphorylation at threonine 172. Enhancing the AMPK pathway reduces vascular inflammation and improves endothelial function, which subsequently contributes to less cardiac remodeling and myocardial dysfunction [168,169,170,171]. Empagliflozin reduced the expression of IL-6, TNF, and MCP-1 in the hearts of diabetic rats in an AMPK-dependent manner [172]. These anti-inflammatory effects also contributed to the inhibition of pro-fibrotic TGF-β/Smad signaling [154], reduced ventricular remodeling, and improved cardiac function [173,174]. AMPK/mTOR signaling [175] modulates autophagy as well, exerting anti-inflammatory effects [176,177]. The improvement in cardiac function by empagliflozin in vivo has been shown to be attributed to autophagosome accumulation and enhanced autophagic flux mediated by the AMPK/mTOR pathway [178].

#### 3.1.7. Effects of SGLT2 Inhibitors on Calcium Homeostasis

The Na+-H+ exchanger isoform 1 (NHE1) is responsible for the adaptation of the heart to intracellular acidosis. Mice overexpressing NHE1 developed cardiac hypertrophy, contractile dysfunction, and heart failure [179]. The overexpression of NHE1 results in increased intracellular Na+ concentrations, leading to enhanced sarcoplasmic reticulum Ca^2+^ loading via the Na+-Ca^2+^ exchanger [179]. SGLT2 inhibitors inhibit NHE1 activity, reduce cardiac cytosolic Na+ and Ca^2+^, and result in coronary relaxation [180].

Empagliflozin also suppresses the NLR family, pyrin domain-containing 3 (NLRP3) inflammasome in macrophages of T2D patients, probably via increased ketones and decreased insulin [181]. Ex vivo experiments showed that high β-hydroxybutyrate and low insulin levels prevent NLRP3 inflammasome activation macrophages with a reduced release of pro-inflammatory cytokines [181].

### 3.2. GLP-1 RA

Although GLP-1 RAs reduce atherosclerotic CV events, their roles in HF prevention and their safety in individuals with clinical HF are uncertain. In addition, the exact mechanism by which these agents improve CV outcomes remains to be clarified.

GLP-1 RAs have been shown to exert a positive chronotropic effect that increases heart rate [182,183]. In patients with HFrEF, an increase in heart rate is associated with worse outcomes [184]. CLP-1 RAs stimulate insulin secretion via activating the cAMP pathway. GLP-1 receptor agonists activate adenylate cyclase, which increases the concentration of intracellular cAMP in cardiac myocytes of the sinoatrial node, where the GLP-1 receptor has been localized, resulting in an increased heart rate [185]. However, the activation of cAMP was shown to worsen HF and increase the risk of death in the PROMISE study [186].

In contrast, liraglutide protects against angiotensin II- and thoracic aorta coarctation-induced pressure overload-induced cardiac hypertrophy and fibrosis. Mechanistic investigation using the overexpression of constitutively active Akt or the knockdown of AMPK demonstrated that liraglutide directly suppresses the Akt pathway and activates the AMPK signaling pathways in cardiomyocytes [187]. Another study showed that continuous infusion of GLP-1 (7-36) for 4 weeks reduced left ventricular stiffness, diastolic dysfunction, and pulmonary congestion and prolonged the survival of mice with HFpEF induced by aortic banding. Further investigation showed the shift in the fuel utilization of heart glucose oxidation [188]. Another study also found that the exenatide analog improves cardiac function, cardiac remodeling, exercise capacity, and survival in rats with chronic heart failure caused by coronary artery ligation [189]. Collectively, GLP1-RA seems to improve HF in either diabetic or non-diabetic rodents through altered energy utilization.

### 3.3. Bariatric and Metabolic Surgery

The mechanism by which bariatric or metabolic surgery exerts a substantially reduced risk of incident of HF is multi-factorial and has been extensively reviewed [190,191]. Obesity increases the preload, afterload, and ventricular wall stress and causes diastolic dysfunction, which could be reversed by the weight loss of bariatric surgery [192,193]. Bariatric surgery also reduces BP, sympathetic tone and heart rate, and systemic inflammation, increases circulating adiponectin, and improves glycemic control, insulin resistance, lipid profiles, and endothelial function [194].

### 3.4. Bromocriptine

**Figure 3:**
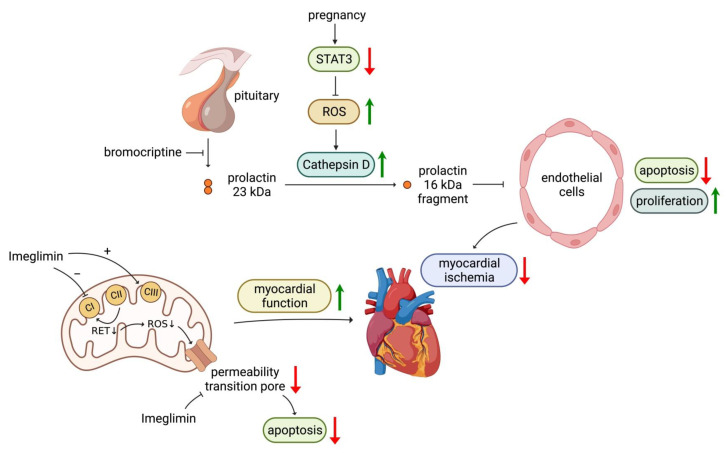
Molecular mechanism of the effect of imeglimin on heart failure and bromocriptine on peripartum cardiomyopathy. CI: respiratory complex I; CII: respiratory complex II; CIII: respiratory complex III; RET: reverse electron transfer; ROS: reactive oxygen species; STAT3: the signal transducer and activator of transcription 3.

Oxidative stress is increased, and the signal transducer and activator of transcription 3 (STAT3) is activated during late pregnancy, which stimulates the expression of cathepsin D in the myocardium, leading to the cleavage of normal 23 kDa prolactin to 16  kDa prolactin fragment [195]. The 16 kDa fragment exerts anti-angiogenic, proapoptotic, and pro-inflammatory effects on the endothelium and cardiomyocyte, leading to vascular and myocardial damage [59,61]. Myocardial STAT3 levels are reduced, and serum levels of activated cathepsin D and 16 kDa prolactin are elevated in patients with peripartum cardiomyopathy (Figure 3) [196].

Mice overexpressing the transcription factor STAT3 were relatively resistant to anthracycline-induced cardiotoxicity via the upregulation of antioxidant enzymes that alleviate oxidative stress in the myocardium [197]. Pregnant mice that lacked STAT3 in their cardiomyocytes had markedly increased oxidative stress and activated cardiac cathepsin D and increased circulating 16 kDa prolactin fragment. These mice displayed features of peripartum cardiomyopathy, which was reversed by bromocriptine, a prolactin inhibitor [196]. These data, together with evidence from clinical trials, support bromocriptine as a therapeutic strategy for peripartum cardiomyopathy.

### 3.5. Imeglimin

Imeglimin improves mitochondrial bioenergetics by improving mitochondrial fatty acid oxidation, rebalancing mitochondrial respiratory chain complex activities, altering mitochondrial phospholipid composition, and decreasing mitochondrial oxidative stress (Figure 3).

Imeglimin modulates respiratory chain activity by partially inhibiting complex I and increasing complex III activity, leading to a decrease in ROS generated from reverse electron flux via complex I without affecting the overall cellular oxygen consumption. Increased mitochondrial ROS production activates the mitochondrial permeability transition pore (mPTP) opening, leading to cell apoptosis. Imeglimin effectively decreases mitochondrial permeability transition pore (mPTP) opening. In contrast to metformin (a weak mitochondrial complex I inhibitor), which potentially suppresses cellular oxygen consumption and increases lactate, imeglimin does not increase lactate production [198]. Although both imeglimin and metformin are complex I inhibitors, their specific mechanisms are different [195].

Imeglimin has been shown to preventHFpEF, a characteristic of diabetic cardiomyopathy, by alleviating the unfolded protein response, reducing downstream inflammation, and reducing lipid peroxidation in the myocardium of mice [66]. Another study showed that acute imeglimin treatment improved coronary endothelial dysfunction and restored coronary relaxation in diabetic rats [67]. Imeglimin markedly reversed the impaired LV function of diabetic rats, including LV end-diastolic pressure LV relaxation constant, and LV end-diastolic pressure volume-relation, LV perfusion, and a parallel reduction in LV ROS production. Of note, the changes occurred regardless of glucose levels [68].

Imeglimin has protective effects on hyperglycemia-induced death of human endothelial cells by preventing the opening of mitochondrial permeability transition pore, cytochrome c release, and cell death [68].

Taken together, despite the lack of RCTs to examine the effect of imeglimin on HF, preclinical studies suggested a potential benefit of imeglimin in diabetic cardiomyopathy, probably due to the central importance of mitochondria in the pathogenesis of HF in T2D patients.

### 3.6. Metformin

Preclinical studies have provided abundant evidence regarding the beneficial effects of metformin on HF in diabetic rodents, and the molecular mechanisms have been previously extensively reviewed [199]. The mechanism behind the cardioprotective effect of metformin is complicated. Metformin is an activator of the 5′ adenosine monophosphate-activated protein kinase (AMPK) pathway. In the heart, the AMPK pathway activates glucose uptake and glycolysis, prevents cardiomyocyte apoptosis, induces autophagy through suppressing the mammalian target of rapamycin (mTOR) signaling, reduces abnormal cytoskeletal proliferation, and increases endothelial nitric oxide synthase (eNOS). It also suppresses inflammation through the activation of Kelch-like ECH-associated protein 1-NF-E2-related factor 2 (Nrf2/Keap1) signaling and subsequent fibrotic response, promotes mitochondrial biogenesis through PGC1α (peroxisome proliferator-activated receptor gamma coactivator 1-alpha), and attenuates cardiac fibrosis by inhibiting the transforming growth factor β (TGFβ)-signaling pathway, thus leading to improved cardiac fuel utilization, attenuated hypertrophy, and reduced fibrosis [200,201,202,203,204,205,206,207,208].

Discoidin domain receptor 2 (DDR2), a collagen receptor tyrosine kinase, regulates the Collagen type I gene expression in the fibroblasts of cardiac and vascular adventitia. By inhibiting TGF-β1/SMAD2/3 signaling, metformin was found to reduce hyperglycemia-induced increases in DDR2 mRNA and protein expression. Thus, metformin regulates these matrix proteins by inhibiting the DDR2-dependent expression of Fibronectin and Collagen type I. DDR2 is a mediator of CV remodeling and a molecular target of metformin. Metformin plays a protective role in the vascular and cardiac fibrosis associated with diabetic cardiomyopathy [209].

However, metformin is a weak inhibitor of mitochondrial complex I, which may decrease the total oxygen utilization of cardiomyocytes and increase lactate generation [197]. Therefore, whether the beneficial effect observed in preclinical studies can be applied to a failing heart should be interpreted cautiously.

### 3.7. Thiazolidinediones (TZD)

**Figure 4:**
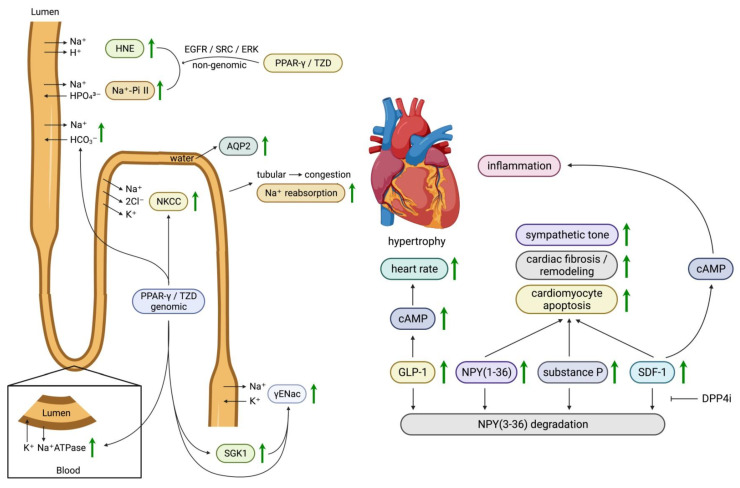
Molecular mechanism of the effect of thiazolidinediones and dipeptidyl peptidase-4 inhibitors on heart failure. HNE: the Na^+^/H^+^ exchanger; NKCC: Na^+^-K^+^-2Cl^−^ co-transporter; Na-Pi2: Na^+^-phosphate cotransporter-2; AQP2: aquaporin2; Na+, K+-ATPase: the sodium–potassium adenosine triphosphatase; PPARγ: peroxisome proliferator-activated receptors γ; TZD: thiazolidinediones; γENaC: epithelial sodium channel γ; SGK1: the serum/glucocorticoid regulated kinase; cAMP: cyclic adenosine monophosphate; GLP-1: glucagon-like peptide receptor; NPY: neuropeptide Y; DDP-4i: dipeptidyl peptidase-4 inhibitors; SDF-1: stromal cell-derived factor 1; CXCR: C-X-C chemokine receptor; Src/EGFR/ERK: Src/epidermal growth factor receptor/extracellular signal-regulated protein kinase.

Nearly all sodium is filtered in the glomerulus, and ~99% of sodium is reabsorbed in the renal tubules. Approximately ~70% of filtered sodium is reabsorbed in the proximal tubule through the NHE3, Na+-amino acid, Na+-phosphate cotransporter-2 (Na-Pi2), Na+-glucose, and Na+-bicarbonate co-transporters. Approximately 20–25% of the sodium is reabsorbed in the ascending limb of Henle’s loop through furosemide-sensitive Na+-K+-2Cl− co-transporter (NKCC), 5–10% in distal convoluted tubules through the thiazide-sensitive Na+-Cl− co-transporter (NCC), and the remaining ~3% in the collecting duct through the amiloride-sensitive epithelial sodium channel (ENaC). Of note, the ENaC in the collecting duct is regulated by aldosterone. After the sodium has been reabsorbed in the tubular cells, the sodium moves further into the peritubular space by sodium–potassium adenosine triphosphatase (Na+, K+-ATPase) throughout all tubule segments (Figure 4).

The mechanism of fluid retention caused by TZD is controversial. Most animal and human studies indicate that renal sodium reabsorption, but not glomerular filtration, is responsible for this phenomenon. Two independent studies [92,210] strongly indicate that TZDs induce more sodium reabsorption by activating γENaC in the collecting ducts. In mice lacking PPARγ in the collecting ducts, neither rosiglitazone nor pioglitazone can induce fluid retention compared with wild-type controls [92,210]. In collecting ductal cells isolated from wild-type mice, pioglitazone stimulated the amiloride-sensitive sodium flux and the expression of γENaC mRNA levels ~10-fold. This effect was thought to be mediated directly by PPARγ, since PPARγ directly binds the peroxisome proliferator response elements (PPREs) with the genomic DNA-encoding γENaC.

The activation of PPARγ was also shown to upregulate γENaC indirectly through serum/glucocorticoid-regulated kinase (SGK1). The intracellular trafficking of ENaC between cytosol and the cell membrane is regulated by SGK1 through regulating proteasomal degradation. Rosiglitazone and pioglitazone increased SGK1 mRNA and protein levels, which was eliminated by the PPARγ antagonist. Furthermore, PPARγ physically interacts with PPREs in the promoter region of the SGK1 gene [211]. However, this hypothesis has been challenged in several studies [212,213,214].

In addition to collecting ducts, the proximal renal tubules and other segments have been shown to be related to TZD-induced edema. In a study of healthy human subjects, pioglitazone reduced sodium excretion and increased sodium reabsorption in the proximal tubules. In rats, the PPARγ agonist upregulated the expression of several genes related to sodium excretion, such as the α-1 subunit of NaK-ATPase, SGK-1, glucocorticoid receptor, and aquaporin 2 (AQP2) [215]. Another study in rats showed that rosiglitazone increased the expression of the α-1 subunit of Na+, K+-ATPase, NHE3, Na-Pi2, and NKCC2 [216]. Interestingly, PPARγ agonism did not increase the expression of γENaC in either study. Collectively, TZD-induced edema is mediated, at least in part, through the PPARγ-mediated upregulation of tubular sodium channels and the peritubular Na+, K+-ATPase in the proximal tubule, ascending loop of Henle, distal convoluted tubules, or the collecting duct.

TZDs were also found to rapidly stimulate the NNE1 and the Na+-bicarbonate co-transporters in the proximal tubule through PPARγ-Src-EGFR-ERK signaling in vitro and in vivo. TZD enhances the direct binding between PPARγ and Src, leading to ERK phosphorylation and the increased expression of downstream sodium transporters. The binding between PPARγ and Src is non-genomic in nature since the overexpression of full-length PPARγ into PPARγ null cells rescues the ERK phosphorylation and NHE1 stimulation. However, the overexpression of the ligand-binding domain (LBD), which lacks a DNA-binding domain, also rescued the NHE1 stimulation and ERK phosphorylation. In contrast, the binding-deficient LBD mutant failed to rescue the NHE1 stimulation or the ERK phosphorylation [216].

### 3.8. Dipeptidyl Peptidase-4 (DPP-4) Inhibitors

The effects of DDP-4 inhibitors on heart failure in preclinical studies are controversial. Alogliptin increases myocardial GLP-1/cAMP levels [101], and prolonged activation of cAMP leads to the exacerbation of HF [186], probably owing to increased heart rates (Figure 4).

In addition to GLP and GIP, DPP-4 also degrades other substrates, including SDF-1, substance P, bradykinin, and NPY. DPP-4 metabolizes NPY1-36 and PYY1-36 (Y1 receptor agonists) to NPY3-36 and PYY3-36 (inactive at Y1 receptors). NPY1-36 and PYY1-36, but not NPY3-36 or PYY3-36, stimulate the proliferation of cardiac fibroblast and stimulate collagen production. DPP4 inhibition enhances the effects of NPY1-36 and PYY1-36 on cardiac fibroblasts [103]. Substance P increases cardiac sympathetic tones. In an RCT of 12 healthy subjects with a crossover design, sitagliptin, in combination with ACE inhibition, enhanced the substance P-mediated increase in heart rate and the vascular release of norepinephrine [104]. SDF-1 triggers cardiac inflammation and fibrosis via binding to the C-X-C chemokine receptor (CXCR), which is reversed by DPP4 inhibitors [97,217,218,219]. SDF-1 [219,220], substance P [221], and NPY [222] also increase sympathetic tone. These change with SDF-1. NPY-Y1 and substance P may aggravate HF through cardiac structural remodeling and cardiomyocyte apoptosis [218,223,224,225].

## 4. Conclusions

Diabetes increases the risk of developing HF. Anti-diabetic therapy with cardio-protective effects for HF reduces the comorbidity of diabetes and HF, and it improves the prognosis of diabetes.

SGLT2 inhibitors were initially found to treat T2D due to their glycosuric effect. However, their CV outcome trials demonstrated an unexpected and significant reduction in HHF [225]. Current evidence further revealed that SGLT2 inhibitors exert beneficial effects on preventing or treating HF in patients with and without diabetes. The possible mechanism of the protective effect of SGLT2 inhibitors on HF includes improvement of mitochondrial energy utilization, the restoration of renal tubular–glomerular feedback with resultant attenuation of renin–angiotensin II–aldosterone activation, diuretic and natriuretic effects, a decrease in sympathetic tone, improvement of mitochondrial calcium homeostasis, and a reduction in myocardial inflammation, oxidative stress, and fibrosis. GLP-1 RA was demonstrated to have a neutral effect on HF by RCTs, probably due to its effect on increasing the heart rate via increasing cAMP. Observational studies have revealed the beneficial effects of bariatric and metabolic surgery on HF. The possible beneficial effect of imeglimin on HF was suggested by preclinical studies, but further clinical evidence is needed. Although abundant preclinical and observational studies support the beneficial effects of metformin on HF, there is limited evidence from RCTs. TZDs increase the risk of HHF through increasing renal tubular sodium reabsorption. Saxagliptin and possibly alogliptin, both DPP4 inhibitors, increase the risk of HHF in RCTs, probably owing to increased circulating vasoactive peptides that impair endothelial function, activate sympathetic tones, and cause cardiac remodeling. Observational studies and RCTs have demonstrated the neutral effects of insulin, sulfonylureas, an alpha-glucosidase inhibitor, and lifestyle interventions on HF in diabetic patients.

Although we seem to be poised on the horizon of exciting new breakthroughs, much knowledge has yet to be obtained before these novel agents are ready for widespread use.

## Data Availability

Not applicable.
